# Investigation on an Innovative Method for High-Speed Low-Damage Micro-Cutting of CFRP Composites with Diamond Dicing Blades

**DOI:** 10.3390/ma11101974

**Published:** 2018-10-13

**Authors:** Zewei Yuan, Jintao Hu, Quan Wen, Kai Cheng, Peng Zheng

**Affiliations:** 1 School of Mechanical Engineering, Shenyang University of Technology, Shenyang 110870, China; jintao_hu@163.com (J.H.); sgd_zhp@163.com (P.Z.); 2 School of Mechanical engineering, Northeastern University, Shenyang 110819, China; wenquan4318@aliyun.com; 3 College of Engineering, Design and Physical Sciences, Brunel University London, London UB8 3PH, UK; Kai.Cheng@brunel.ac.uk

**Keywords:** CFRP composites, diamond dicing blades, micro cutting, low-damage surface, surface roughness

## Abstract

This paper presents an innovative method for high-speed micro-cutting of carbon fiber reinforced plastics (CFRP). It employs a diamond dicing blade for micromachining applications, with a thickness of about 200 μm and rotational speeds up to 30,000 rpm so as to meet the low-damage surface integrity requirements. The process parameters, cutting damage, surface roughness, and the spindle vibration were thoroughly investigated to evaluate and validate the method. The results indicate that a high cutting speed up to 76 m/s not only remarkably increases the rigidity of an ultra-thin dicing blade, but also decreases the cutting depth per diamond grit to below 10 nm, both of which are very conducive to obtaining a very fine machined surface of about Ra 0.025 μm, with no obvious damage, such as delamination, burrs, and fiber pull out. The serious spindle vibration limits the rotational speed to increase further, and the rotational speed of 25,000 rpm achieves the best fine machined surface. Furthermore, unlike most research results of the drilling and milling method, the proposed micro-cutting method obtains the maximum cutting current and surface roughness when cutting at 0° fiber orientation, while obtaining a minimum cutting current and surface roughness when cutting at 90° fiber orientation. The metal-bonded dicing blade achieves smaller surface roughness than the resin-bonded dicing blade. This paper also discusses the cutting mechanism by investigating the morphology of the machined surface and concludes that the micro breakage and plastic-flow in local regions of fibers and resin are the main material removal mechanisms for dicing CFRP composites with a diamond abrasive blade.

## 1. Introduction

Carbon fiber reinforced plastic (CFRP) composites, created by blending carbon fibers into plastic resin, have a variety of desirable properties including low density, high stiffness to weight ratio, excellent fatigue, corrosion and wear resistance, outstanding toughness and damage tolerance, high dimensional stability, excellent chemical resistance, and low friction coefficient, thermal expansion, and electrical conductivity, which cannot be obtained from conventional metals such as steel and aluminum [1,2,3]. Due to the combination of these significantly distinguished mechanical and thermal properties, CFRP composites have been widely used in place of metals in many different applications, such as aerospace and commercial aircraft, marine, medical prosthesis, sports goods, robot arms, and racing cars [4,5]. In order to meet dimensional tolerance, surface quality, and other functional requirements in these application fields, secondary machining processes such as milling, drilling, grinding, trimming, and waterjet cutting are usually necessary to carry out during the manufacturing of components from CFRP composites [6,7,8]. However, CFRP composites are a typical difficult-to-machine material due to its heterogeneity, anisotropy of mechanical properties, and low thermal conductivity [9]. It is difficult for traditional methods to machine CFRP composite with fine quality. During machining, very rapid cutting tool wear development is experienced [10]. Additionally, serious surface integrity damage is often produced in the machined CFRP parts, such as delamination, burrs, fiber pull out, fiber matrix debonding, and inadequate surface roughness of the cut walls, which will remarkably reduce the mechanical performance of the CFRP parts [11,12,13].

Many researchers identified the type and orientation of fiber, cutting parameters, and tool geometry as decisive factors for the cutting process [14,15]. They researched the cutting parameters including cutting speed and feed rate, the tool materials and geometry, fiber orientation angle, the cutting temperature, and cooling style to improve the machining quality of CFRP [6,16]. In addition, the evaluation of cutting force and hole quality, such as delamination and other damage, is often conducted [17,18]. Orthogonal cutting and macroscopic or microscopic numerical simulations are effective methods to investigate the material removal mechanisms and fundamental failure modes [19,20]. These investigations make significant improvement in reducing damage size such as delamination, fiber pull out, and the surface roughness of the machined walls. For example, the sizes of delamination and burrs have been controlled in millimetric or submillimetric ranges. The surface roughness of the drilling or milling walls can be reduced to within the dimension of several micrometers. Even so, the machining quality cannot meet the requirements of CFRP composites in many new applications such as automobiles, laptop computers, space shuttles, and satellites [21]. These applications usually pursue submicrometer, or even better, cutting damage for CFRP composites. One of most important reasons to produce machining damage is rapid tool wear caused by abrasives of CFRP composites, resulting in a decrease of tool cutting life and an increase of manufacturing costs [22]. After the tool wears out, it is easy to generate vibration during the machining process, the cutting performance of the material is degraded, and the heat energy accumulation increases during the cutting process, so that the tool accelerates wear [23]. Therefore, diamond coated carbide, diamond abrasive, and polycrystalline diamond tools are usually employed to reach a sufficient tool lifetime and to reduce the machining damage [24,25,26]. Many recent studies have demonstrated that diamond abrasive tools can generate a low-damage surface with average surface roughness of about Ra 1.5 μm and higher dimensional accuracy [27,28]. Later, rotary ultrasonic machining produced even better surface quality with a surface roughness of about Ra 1.0 μm [29,30,31]. Other research about the machining of CFRP composites indicated that high-speed machining with spindle speeds up to 60,000–80,000 rpm could also generate good machining surface and high machining efficiency, mainly due to the remarkable reduction of cutting forces [32,33]. These studies proved that high-speed cutting with abrasive tools generates a better surface intensity for CFRP composites.

Besides conventional methods, non-conventional machining methods including water jet cutting, abrasive jet cutting, electrical discharge machining, and laser machining are common methods for cutting CFRP composites and other difficult-to-cut materials [34,35,36]. However, disadvantages of water jet cutting are the elaborate microfiltration before, and the treatment and disposal of water after, machining. As an alternative tool for the cutting of CFRP composites, laser cutting has non-contact, dry, and abrasionless advantages [37]. Its heat affected zone can be controlled in the dimension of 10–200 μm, depending mainly on the intensity of the laser beam, the intensity profile, and the thickness of laminates [38,39].

Diamond blades, created by embedding diamond particles in a metal matrix or brazing diamond particle onto the borders of a steel core, can increase significantly the life of cutting tools, especially for high-speed machining hard-to-cut materials. It was first widely designed to cut hard or abrasive materials, such as concrete, marble, stone, and ceramics, when its thickness and the size of diamond particles are large [40,41,42]. Then, ultra-thin and high-precision diamond blades are developed to use in slicing, dicing wafer, cut-off, singulation, grooving, slotting, cross sectioning, sample preparation, gang sawing, slabbing, and fine cutting applications. As one of the most common methods for precision cutting, bladed dicing is characterized by using abrasive thin blades as the cutting element [43,44]. The dicing blade is fixed on a high-speed spindle normally supported by aerostatic or ceramic bearings. The blade can reach up to 60,000 rpm and a blade with a diameter of 51 mm would reach a peripheral cutting speed at about 160 m/s. Thus, a high cutting speed and the well-wearing resistance of diamond abrasives are an effective way to reduce the machining damage of nature fiber composites and CFRP composites [45,46]. Therefore, diamond dicing blades are novel to use in the micro cutting of CFRP composites, especially with micron-sized or submicron-sized machining damage and surface roughness.

To satisfy the high machining quality demands in new applications of automobiles, laptop computers, space shuttles, and satellites, this work proposed a new high-speed micro-cutting method for CFRP composites. It employed diamond abrasive dicing blades with a thickness of about 200 μm to cut CFRP laminates with spindle speeds of up to 40,000 rpm. The cutting parameters, cutting damage, surface roughness, and the influence of spindle vibration were analyzed in detail. Additionally, the cutting mechanisms were also discussed with the help of a scratching experiment on the UMT-TriboLab platform (Bruker, Madison, WI, USA).

## 2. Methodology and Experiments of Micro-Cutting CFRP Composites

**Figure 1:**
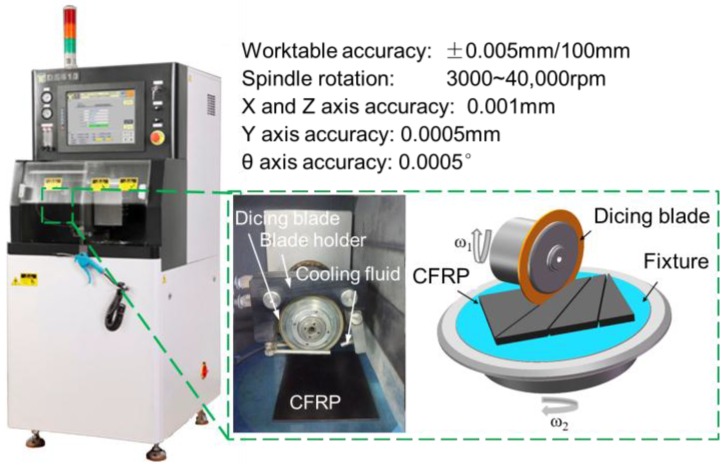
Slicing machine and its parameters. CFRP: carbon fiber reinforced plastic.

Basically, the quality of kerf depends on the combination of the material being worked upon and the parameters of the cutting process, cooling system, feed speed, type of blade, and peripheral speed [47]. However, for the ultraprecision dicing of CFRP composites, the failure mechanism is more complex than hard and brittle materials because of its heterogeneity, anisotropy of mechanical properties, and low thermal conductivity. Therefore, the comprehensive factors that affect cutting damage should be investigated. As shown in Figure 1, the dicing machine has an accuracy as small as 1 μm. A CFRP laminate was fixed on a ceramic sucker so that it undergoes uniform load when it is chucked.

**Figure 2:**
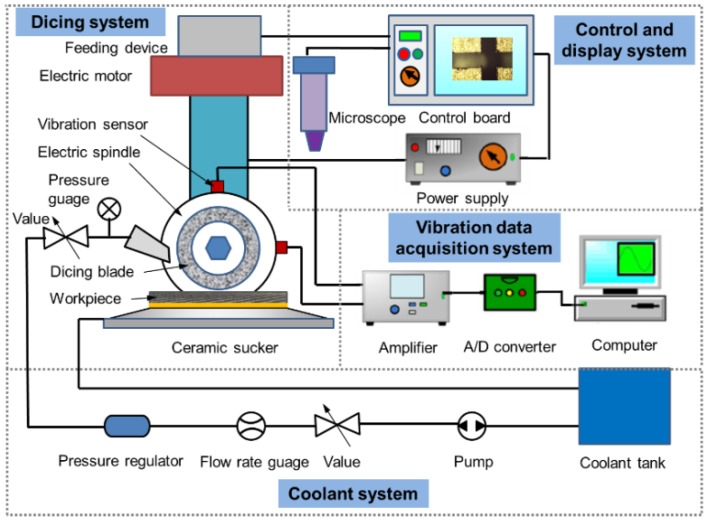
Schematic diagram of dicing system for CFRP composites.

As illustrated in Figure 2, the control and display system in the machine was used to locate and observe the cutting kerf. It is easy to calculate the wear amount of the dicing blade by twice tool setting before and after the dicing process. Due to the sensitivity of CFRP composites to machining temperature, the dicing blade and cutting region of the workpiece were cooled by the coolant system during the dicing process. Hereby, deionized water was used as coolant. Furthermore, the vibration of the spindle has an important influence on the micro-cutting damage of CFRP composites, so a vibration data acquisition system was fixed onto the dicing machine. Two vibration sensors were adhered onto the surface of spindle with an angle of 90° to perceive the vertical and horizontal vibrations of the spindle. Because it is difficult to fix a dynamometer onto the cutting machine, the cutting current was measured to reflect the variation of the cutting force.

**Figure 3:**
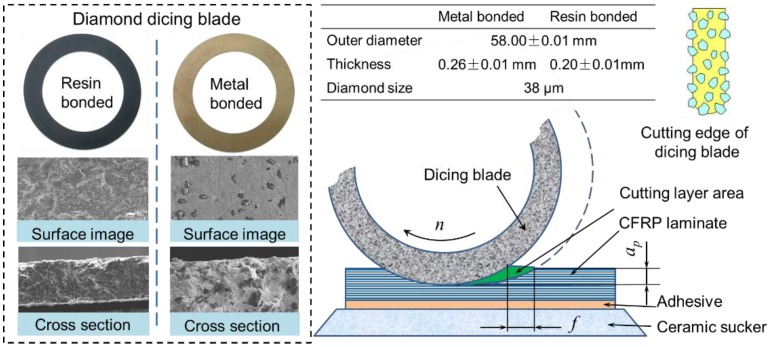
Structure of dicing blade and schematic diagram of cutting process.

As the cutting tool, the dicing blade has a remarkable influence on the cutting quality. Its preparation method is a key factor for machining performance. The complex preparation process of the dicing blade includes mixing powders, filling powders into the graphite die, sintering powders, reducing thickness, and dressing the shape. According to the different matrices in the dicing blade, the precision dicing blades are divided into three types: metal-sintered diamond blades, resin-bonded diamond blades, and electroplated nickel bond diamond blades. Metal-sintered diamond blades have a higher blade lifetime due to the higher thermal conductivity and better mechanical properties of the binder material. Resin-bonded diamond blades are used in applications that require a smooth surface finish and a minimum amount of chipping. Electroplated nickel bond diamond blades have a high diamond concentration and give a freer, faster cutting action with minimum heat generation. In these cutting experiments, two types of diamond dicing blade, including metal-bonded dicing blade and resin-bonded dicing blade, were used for cutting CFRP composites. The thicknesses of the two types of dicing blades were 0.26 ± 0.01 mm and 0.20 ± 0.01 mm, respectively. The diamond size of dicing blades was about 38 μm, which is much smaller than most of the electroplated diamond tools used for drilling CFRP laminates. The cross-section structure and the diamond abrasive distribution of the used dicing blades are shown in Figure 3. It can be seen that the diamond abrasives distribute uniformly in the blade matrix and has a sharp edge. If the diamond abrasives in the blade surface wear, they will fall off the blade under the action of cutting force, which is larger than the bonding force of diamond grits with the matrix. Thus, the diamond blades have better self-sharpening properties in comparison with PCD (polycrystalline diamond) tools or diamond-coated cemented carbide tools. The cutting layer area is also much smaller than that of traditional grinding. Thus, the diamond dicing blade may generate a much smaller cutting force than traditional methods, which can result in small or free machining damage for CFRP laminates.

**Figure 4:**
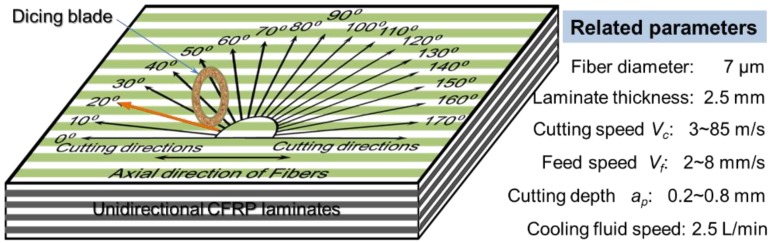
Schematic diagram of cutting direction and cutting parameters.

In order to demonstrate the influence of the cutting parameters and fiber orientations on cutting quality, the cutting experiments with different fiber orientations and cutting speeds were conducted on the dicing machine (Heyan Technology, Shenyang, China) as illustrated in Figure 4. The rotational speed of the spindle varied from 1000 to 28,000 rpm, in correspondence with the cutting speed from 3 to 85 m/s. The feed speed and cutting depth were 2~4 mm/s and 0.3~0.6 mm, respectively. It can be observed that the cutting speed is obviously larger than that of most reported machining methods for CFRP composites. The specimens used for cutting in these experiments were unidirectional T300 CFRP laminates (Shenyang Aircraft Corporation, Shenyang, China). After the cutting process, the sliced sections of CFRP laminates were observed with an optical microscope (Shanghai Optical Instrument Co., LTD., Shanghai, China) and scanning electron microscope (SEM, Hitachi High-Technologies Corporation, Tokyo, Japan) to characterize the dicing damage in different dicing conditions. 

## 3. Results, Analysis, and Discussion

### 3.1. Influence of Different Cutting Speeds on Cutting Quality

**Figure 5:**
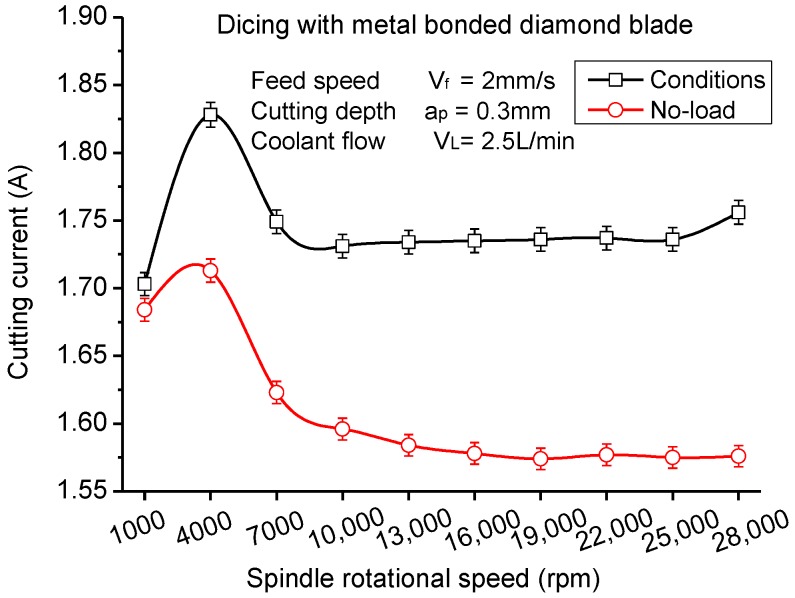
Maximum cutting current varying with spindle rotational speed.

Just like other nature fiber composites with heterogeneous and anisotropic properties, the cutting quality of CFRP composites is also very sensitive to the cutting speed. In low cutting speeds, the fibers embedded in the matrix are easy to bend so as to cause large machining damage, such as delamination and debonding [46]. High machining speeds over 40 m/s can generate good surface quality for wood and other fiber reinforced composites [48]. In this study, the cutting current of the spindle and surface roughness are observed to characterize the influence of cutting speed. Many studies on the relation between the cutting force and the motor current [49] proved that the change of spindle motor current value reflects the cutting force well, and cutting force obtained by measuring the spindle motor current is reasonable and feasible. Due to the limited working space in the dicing machine, it is difficult to place a dynamometer onto the worktable to detect the cutting force. Thus, in this experiment, the cutting current obtained from the spindle motor was employed to study the change of cutting force with different cutting conditions. As illustrated in Figure 5, the cutting current of the spindle motor first increased with the rotational speed of the spindle and then decreased under the condition of no-load. The cutting current under the cutting condition has a similar trend of that of no-load. The difference is that there is an obvious increase when the spindle rotational speed changes from 25,000 to 28,000 rpm under the cutting condition. It indicates that under rotational speeds higher than 25,000 rpm, the cutting resistance increases enormously. High cutting current in the rotational speed of 4000 rpm attributes to the self-excited chatter vibrations of the spindle. Under the condition of low rotational speeds, the cutting layer area is larger than that of high rotational speeds. Cutting force and cutting current therefore increases in low rotational speeds.

**Figure 6:**
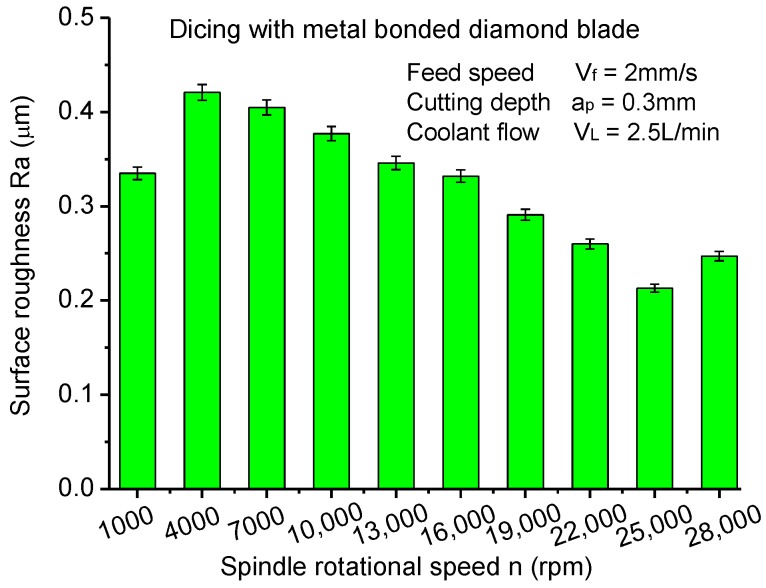
Surface roughness varying with spindle rotational speed.

As presented in Figure 6, the surface roughness of machined CFRP laminates appears to have the same tendency as the cutting current, which illustrates that there is a significant correlation between cutting current and surface roughness. According to previous studies, the cutting current can reflect the cutting force reasonably. Thus, small cutting forces will generate lower surface roughness. It can conclude that CFRP laminates present the best cutting quality at the rotationl speed of 25,000 rpm where the spindle motor runs steadily. Too high rotational speeds of the spindle are not beneficial in improving the dicing quality of CFRP laminates. Meanwhile, the deformation of the dicing blade and vibration of the spindle in low rotational speeds increases the surface roughness of CFRP laminates.

Because the thickness of the dicing blade is very thin in comparison with the traditional grinding wheel, it is easy to deform or even break under the cutting force. In order to prevent it breaking, increasing the rotational speed is an effective method to shift the dynamic stiffness of the dicing blade. Therefore, the thin dicing blade does not deform like paper during the cutting process. If the dicing blade is regarded as a hollow disk with a rectangular section, according to the theory of solid mechanics, the maximum stress and displacement of the dicing blade can be calculated with the following equations.

(1) $$\sigma_{r}=\frac{3+\mu }{8}\rho \omega^{2}\left(r_{0}^{2}+r_{i}^{2}-r^{2}-\frac{r_{0}^{2}r_{i}^{2}}{r^{2}}\right),$$

(2) $$\sigma_{\tau }=\frac{3+\mu }{8}\rho \omega^{2}\left(r_{0}^{2}+r_{i}^{2}-\frac{1+3\mu }{1-3\mu }r^{2}+\frac{r_{0}^{2}r_{i}^{2}}{r^{2}}\right),$$

(3) $$u=\frac{1-\mu^{2}}{8E}\sigma \omega^{2}\left[\frac{3+\mu }{1+\mu }\left(r_{0}^{2}+r_{i}^{2}\right)r+\left(\frac{3+\mu }{1-\mu }\right)\frac{r_{0}^{2}r_{i}^{2}}{r^{2}}-r^{3}\right],$$

where, $r=\sqrt{r_{0}r_{i}}$, *σ_r_*, *σ_τ_* and *u* are radial stress, circumferential stress, and displacement, respectively. *μ*, *ρ*, and *E* are the Poisson’s ratio, density, and elasticity modulus of the dicing blade, respectively. Because the diamond abrasive dicing blade is made of typical heterogenetic materials, it is difficult to calculate the above parameters accurately. Thus, in this study the average values of Poisson’s ratio, density, and elasticity modulus are estimated as 0.22, 5.1 × 10^3^ kg/m^3^, and 128 GPa in consideration of the proportion of diamond abrasive and matrix.

**Figure 7:**
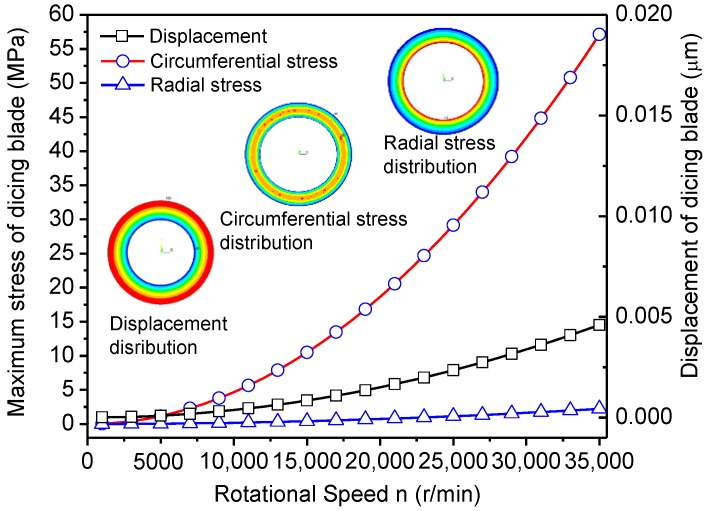
Maximum stress and displacement of the dicing blade with different rotational speed.

Figure 7 shows the simulation results of circumferential stress, radial stress, and displacement of the dicing blade. With the increase of rotational speed, circumferential stress and displacement of dicing blade increase remarkably. High circumferential stress spreads the dicing blade to ensure that the dicing blade has enough rigidity, although it is so thin like paper. It effectively prevents the dicing blade from large deformations under the interactions of the cutting force. That is the reason why the cutting quality of CFRP composites is better at high rotational speeds. Also, it may be a precondition for micro-cutting CFRP laminates with an ultra-thin dicing blade.

### 3.2. Influence of Spindle Vibration on Cutting Quality of CFRP Laminates

**Figure 8:**
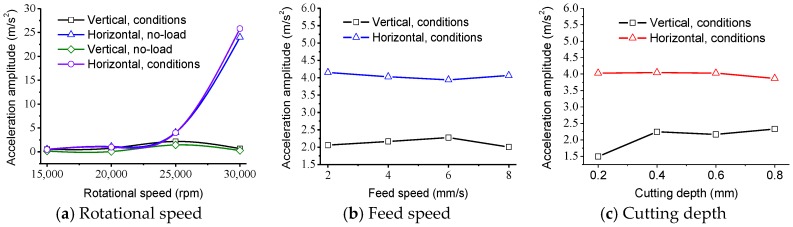
Vibration acceleration amplitude varying with cutting parameters in time domain.

The main mechanisms generating excessive cutting tool vibration can be classified into three types: regenerative, mode coupling, and velocity-dependent [50]. Velocity-dependent mainly originates from the self-excited chatter vibrations of spindle. When the rotational speed is about 4000 rpm, the resonance of the spindle drives the large vibration of the cutting tool, which therefore results in large cutting surface roughness. The regenerative vibration has a simple relationship between the cutting force coefficient, dynamic stiffness of the structure, spindle speed, and depth of cut [51]. The vibration stability has been attributed to either the change in the direction of cutting speed or the cutting force between the cutting tool and the finished workpiece. During the cutting process with the dicing blade, the cutting speed is located in the direction normal to the axis of the spindle. Furthermore, the cutting force can be divided into two component forces in vertical and horizontal directions. Thus, the vibrations in the vertical and horizontal directions are obvious. When the rotational speed increases to 28,000 rpm from 25,000 rpm, the vibration in the horizontal direction has a significant increase, as shown in Figure 8. It demonstrates that the large surface roughness and cutting current is mainly caused by the vibration in the horizontal direction. The vibration of the spindle in the vertical direction is small due to the weight of the spindle and the load shift in the stiffness of the spindle in the vertical direction. Besides, the change of the cutting force in the horizontal direction due to the heterogeneity between the fiber and resin matrix can also be attributed to the large vibration. Other cutting parameters, such as feed speed and cutting depth, have a small effect on the vibration amplitude of the spindle. As shown in Figure 8c, there is a slight increase of vibration amplitude in the vertical direction when the cutting depth changes from 0.2 to 0.8 mm, while the vibration amplitude of the spindle in the horizontal direction keeps a steady change varying with the cutting parameters.

**Figure 9:**
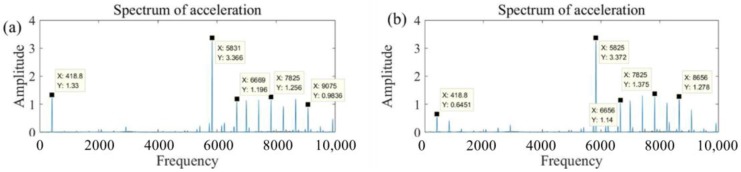

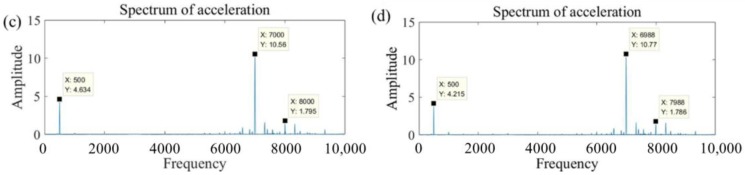
Vibration acceleration spectrum diagram with different conditions in frequency domain. (**a**) With a rotational speed of 25,000 rpm and no-load condition; (**b**) with a rotational speed of 25,000 rpm and conditions, (**c**) with a rotational speed of 30,000 rpm and no-load condition; (**d**) with a rotational speed of 30,000 rpm and conditions.

Figure 9 illustrates the vibration acceleration spectrum diagram with different conditions in frequency domain. As can be seen from the figures, the vibration amplitudes under the condition of cutting are a little larger than the vibration amplitudes under the condition of no-load. It indicates that the cutting force has a weak effect on the vibration of the spindle because the cutting force generated by the ultra-thin dicing blade is very small at high rotational speeds. However, it should be noted that the vibration amplitudes under the rotational speed of 30,000 rpm are apparently more serious than that of 25,000 rpm. The serious vibration also results in larger surface roughness of CFRP laminates after cutting with the dicing blade. Moreover, there is no new vibration frequency to create by comparing cutting conditions with the no-load condition. The vibration during the cutting process mainly derives from the spindle motor and the dicing machine itself but not the cutting force. The promotion of a small cutting force generated by the dicing blade to the vibration of the spindle is very weak, although the vibration plays a remarkable role on the surface roughness of CFRP laminates.

### 3.3. Influence of Fiber Orientations on Dicing Quality

**Figure 10:**
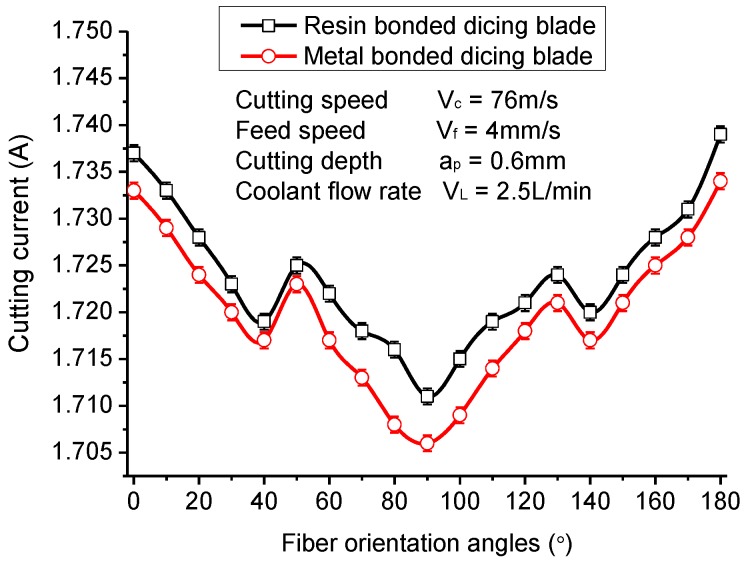
Maximum cutting current varying with fiber orientation.

The fiber orientations have remarkable influence on the cutting current and surface quality due to the outstanding anisotropy of CFRP laminates. Figure 10 shows the maximum cutting current of the spindle motor when different fiber orientation laminates are cut with the resin-bonded dicing blade and the metal-bonded dicing blade. It can be noted that the cutting current of the spindle motor with the metal-bonded dicing blade are overall smaller than that of with the resin-bonded dicing blade, which is different from the cutting of regular brittle materials, such as glass. When cutting brittle materials, the cutting force with a resin-bonded blade is usually smaller than the metal-bonded blade due to the small bonding force of diamond grits with resin matrix. For cutting CFRP composites in this experiment, the poor thermal conductivity of the resin-bonded blade and the CFRP workpiece led to the increase in cutting temperature and the softening of the resin, which results in shifting the cutting force. Therefore, the large cutting force generates a larger cutting current with the resin-bonded blade and causes more serious wear to the dicing blade. It can be verified by the results of the cutting experiments. The wear amounts of the resin-bonded blade in the diameter direction is about 0.17 mm per cutting 10 m, while the wear amounts of the metal-bonded blade is about 0.13 mm per cutting 10 m.

The cutting currents of the spindle motor present an overall V-shape tendency when the fiber orientation angle changes from 0° to 180°. The minimum cutting current occurs at the fiber orientation of 90°. That is to say that cutting along with fiber orientation can generate a maximum cutting current, and cutting perpendicular to fiber orientation can generate a minimum cutting current. Moreover, there are two low points at the fiber orientations of about 40° and 140°. All these tendencies are contrary to most reported results of milling and orthogonal cutting of CFRP composites [19,20]. Usually the cutting force will increase with the fiber orientation as it changes from 0° to 90°. The differences can be attributed to the different cutting mechanisms between dicing and milling or orthogonal cutting. During the milling and orthogonal cutting, the feed rate or feed per tooth is far larger than the diameter of a fiber. Many fibers, together with the resin matrix, are cut down at one time [52]. It results in the carbon fibers and resin matrix undergoing serious deformation under the large cutting force generated by the large tool edge radius. However, for dicing CFRP composites with the diamond dicing blade, the feed rate can be calculated at about 9.6 μm/r. Additionally, there are more than one thousand diamond grits that participate in the cutting in the circumference of a dicing blade. Thus, the feed rate per grit is no more than 0.0096 μm, which is far smaller than the diameter of carbon fiber. With the small cutting depth, the cutting action or material removal only occurs at the local regions of carbon fiber or resin matrix. It cannot cause large deformation to the fibers or resin matrix. Thus, the cutting damage is limited in a small region correspondingly and a fine machined surface is obtained.

**Figure 11:**
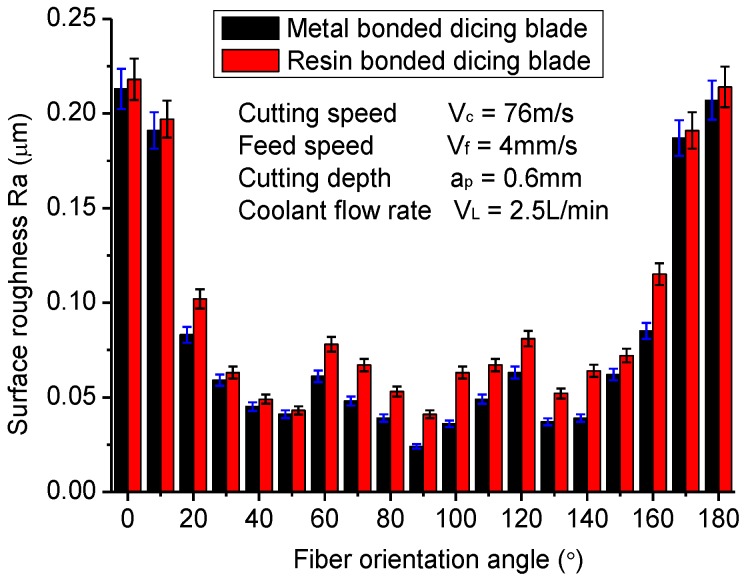
Surface roughness varying with fiber orientation.

Figure 11 gives the surface roughness of machined CFRP composites varying with the fiber orientation. It is interesting that the tendency of the surface roughness changing with fiber orientation is very similar to that of the cutting current. It illustrates that the surface roughness of diced CFRP composites has a significant correlation with cutting force. When the cutting direction is vertical to fiber orientation, the surface roughness of machined CFRP composites is smallest, which is about Ra 0.025 μm, better than most reported experimental results with drilling, milling, or grinding methods. The fiber orientations of 50° and 130° also achieve a relatively better diced surface, while the surface roughness is worst when the fiber orientation is 0° or 180°. These tendencies are also different from traditional milling and drilling processes just as is the cutting current of the spindle motor. Furthermore, the metal-bonded dicing blade generates better surface roughness than the resin-bonded dicing blade, mainly due to its low cutting force and good thermal conductivity. 

**Figure 12:**
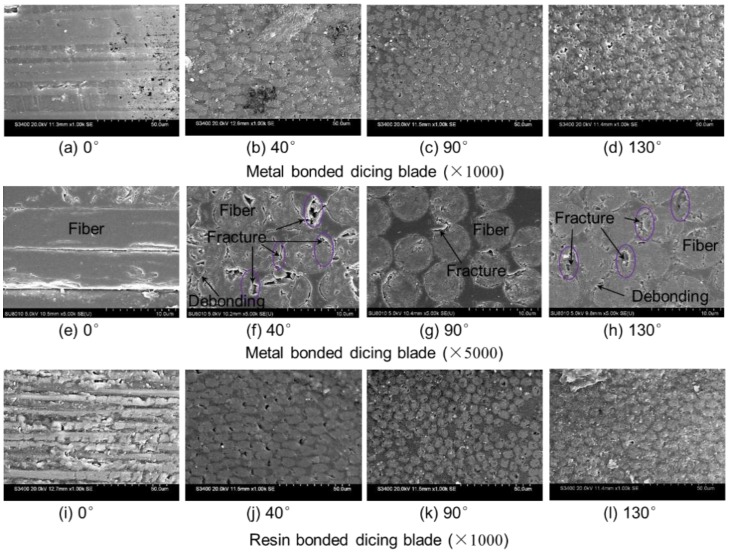
Surface morphologies of CFRP composites after dicing with different fiber orientation.

In order to address the reason of the above tendency of surface roughness, surface morphologies of diced CFRP composites with different fiber orientations are investigated, as shown in Figure 12. As can be seen from the figures, the surface qualities of diced CFRP composites with the metal-bonded dicing blade are better than that of the resin-bonded dicing blade. According to the observations of cutting morphologies, one of main reasons causing large surface roughness with the resin-bonded dicing blade is that there is some molten resin on the machined surface of CFRP composites. It reflects that the high dicing temperature occurs during this process. Under the high temperature, a lot of molten resin adheres to the dicing blade and the machined surface, which not only reduces the cutting performance of the dicing blade, but also increases the machined surface roughness of CFRP composites and the cutting force. In view of cutting CFRP composites with 0° fiber orientations as shown in Figure 12a,e, the fibers have been cut off along their axis. The machined surface of the left-half sections presents very good quality in a small region. But not all fibers are strictly embedded in the direction parallel to the machining surface. The fibers not well embedded in the resin matrix will debond from the matrix due to the poor bonding strength of half fiber. The left grooves cause a larger measured surface roughness than other fiber orientations. When the CFRP composite is sliced at 90° fiber orientation, the fibers are cut smoothly due to the well fixation of fibers in the matrix, according to Figure 12c,g. They have not undergone serious deformation under the cutting force, like milling or drilling. During the milling or drilling process, bunches of fibers bend under the cutting force and finally break to form a zigzag or uneven cutting section [22]. Fiber-tensile failure and matrix-compression failure are normally used to explain the cutting mechanism for these methods [53]. The microtopography of the machined surface of CFRP with the dicing blade shows its cutting mechanisms are different from that of milling and drilling. Furthermore, as shown in Figure 12b,f,d,h, there are lot of micro fractures on the machined section of CFRP composites when cutting with 40° fiber orientation and 130° fiber orientation, although the machined surface is very even. Most of these micro fractures are located at a right position of the fiber section when cutting with 40° fiber orientation, and at a left position of the fiber section when cutting with 130° fiber orientation. All these positions are at the wedge tip of fiber ends, and the material strength is poor. It is easy for the materials at the tip of the fiber to break off under the actions of cutting force and vibration. Thus, the surface roughness cutting at 40° or 130° fiber orientation is larger than that of 90° fiber orientation. Besides the micro fracture, it should be noted that there are slight fiber debondings with the resin matrix when cutting with 40° and 130° fiber orientations. It means that the fibers bend towards the fiber bundle extrusion. But this debonding between fiber and matrix is far weaker than other methods. That is to say that a very smooth machined surface can be obtained when the cutting depth is smaller than the diameter of fibers. No serious material damage, such as delamination, burrs, fiber pull out, are detected, although obtained surface roughness is larger than cutting traditional brittle materials.

### 3.4. Scratching CFRP Composites with Diamond Indenter

**Figure 13:**
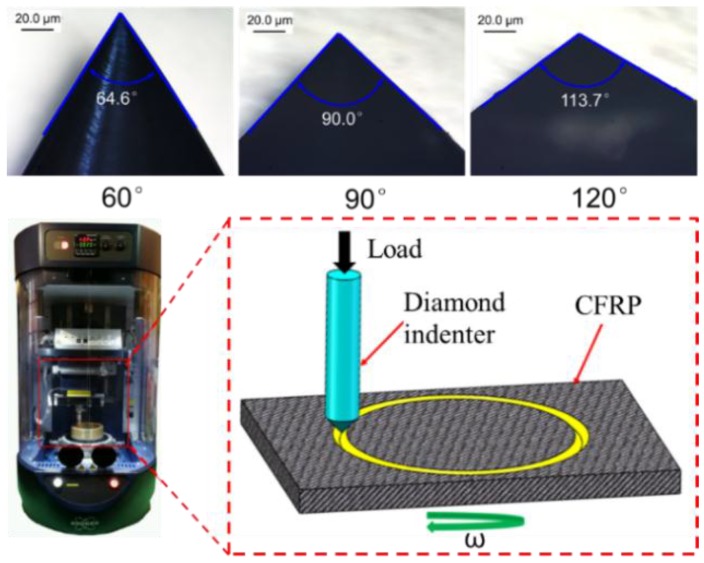
Schematic diagram of scratching CFRP composites with diamond indenter.

**Figure 14:**
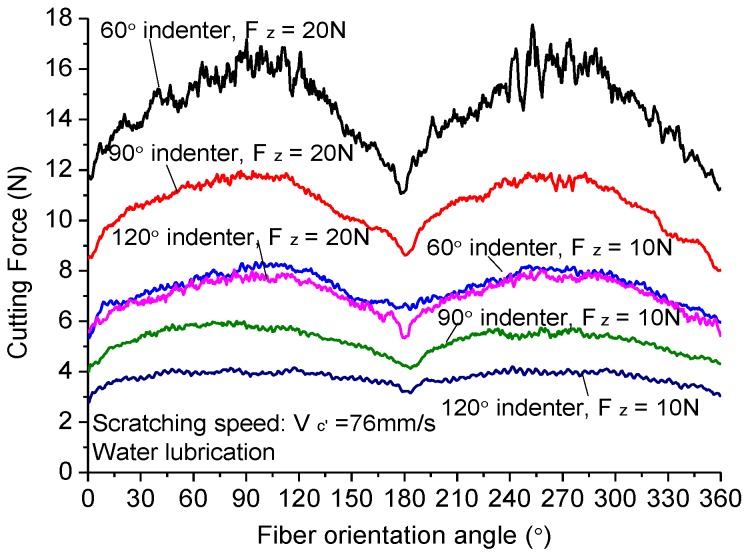
Scratching force varying with fiber orientation.

In order to reveal the cutting mechanism of dicing CFRP composites, the scratching experiments of CFRP composites with a diamond indenter were conducted on the UMT-TriboLab platform to compare the machined quality and cutting force with different fiber orientations and indenter angles, as shown in Figure 13. Three indenters with apical angles of 60°, 90°, and 120° were chosen to reflect the sharpness of diamond abrasive grits in the dicing blade. The sharpness of diamond indenters was well kept before the scratching experiment. Figure 14 shows the scratching force changing with the fiber orientations and the scratching load. Under the same load force, the indenter with apical angle of 60° generates the highest scratching force. The sharp tip allows it to indent into CFRP composites at a higher depth. More carbon fibers take part in scratching, so the scratching force is largest. The indenter with apical angle of 120° has a blunt point. It is different to indent into the CFRP. In most circumstances, it scratches on the surface of CFRP composites but does not cut the fibers. Thus, the scratching force is small. Besides, under the large indent force, the carbon fibers often break off instead of being cut off. Moreover, fiber orientations have remarkable impact on the scratching force. As seen from the figure, the scratching force increases with the increase of fiber orientation from 0° to 90° and then goes down with the increase of fiber orientation from 90° to 120°. When scratching direction is perpendicular to fiber orientation, the scratching force is largest, which is different from the trend of dicing CFRP composites with the diamond blade. It indicates the two cutting actions are of different machining mechanisms. The scratching mechanism is similar to the milling and drilling of CFRP composites.

**Figure 15:**
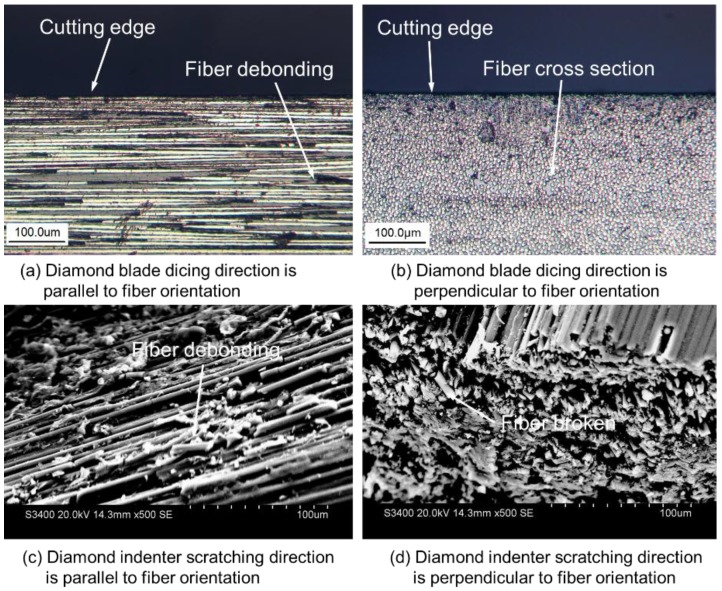
Morphologies of CFRP composites obtained by different cutting methods.

As shown in Figure 15, the comparisons between the morphologies of CFRP composites obtained with the dicing method and scratching identify that the dicing method with a diamond blade can provide much better surface quality of CFRP composites than traditional cutting. During the dicing process with a diamond blade, the carbon fibers were even cut along with its axis without breaking the carbon fibers. Only when the fiber orientation is not parallel to the dicing surface, the fibers will debond from the matrix under the cutting force of diamond grits. Thus, there are many debonding grooves left by carbon fibers, which is the main reason why the surface roughness is largest when the dicing direction is parallel to the fiber orientation. When the dicing direction is perpendicular to the fiber orientation as seen in Figure 15b, carbon fibers embedded well in the resin matrix are difficult to deform during the high-speed cutting process. Thus, carbon fibers are cut orderly. The surface roughness in this fiber direction is therefore best. In comparison with diamond blade dicing, the morphologies of CFRP composites obtained by the diamond indenter are much poorer, as presented in Figure 15c,d. Fiber bundles debonding or breaking are the main cutting mechanisms for diamond indenter scratching. This cutting style dominates that the surface roughness of machined CFRP composites is larger than the scales of carbon fibers. 

### 3.5. Cutting Mechanism of CFRP Composites with Diamond Blade

**Figure 16:**
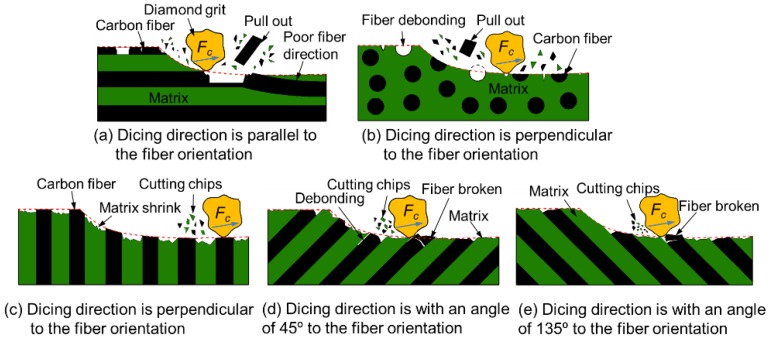
Schematic diagram of dicing mechanism with different fiber orientations.

According to the above analysis, the cutting mechanism of CFRP composites with a dicing blade can be briefly concluded as illustrated in Figure 16. The cutting depth per diamond grit (about 10 nm) during the dicing process is much smaller than the diameter of the carbon fiber (about 7 μm). Under this machining condition, the cutting force is too small to cause the serious deformation or breaking off of bulk fiber bundles. The cutting force of diamond grits only acts in a small region of carbon fiber or resin matrix. Thus, serious macro damages, such as delamination, burrs, and fiber pull out, are prevented from occurring in the dicing method, which is different from traditional machining methods [54]. As far as fiber orientation is concerned, when the dicing direction is parallel to the fiber orientation, fibers on the surface of CFRP composites are not embedded in matrix entirely. It is easy for them to debond from the resin matrix under the scratching action of diamond grits. The residual debonding grooves are the main reason in causing the increase of surface roughness. When the cutting direction is perpendicular to the fiber orientation, carbon fibers are fixed well in the resin matrix. Only the ends of fibers are cut by diamond grits. It is difficult for fibers to deform under the condition of a very small cutting depth. Thus, the cutting surfaces are very smooth. Under these circumstances, the shrinking of resin after the dicing process is the main cause in increasing the surface roughness of machined CFRP composites due to the decrease in temperature. When the dicing direction is at an angle of 45° or 135° to the fiber orientation, the tips of fibers are easy to break off under the cutting force of diamond grits because of poor support. Thus, the surface roughness is larger than that of the dicing direction perpendicular to the fiber orientation. According to the study of Bifano, plastic-flow is the predominant material removal mechanism for grinding brittle materials when grinding infeed rates are controlled as small as several nanometers per grinding wheel revolution [55]. Although somewhat brittle, the cross-section surface of carbon fiber finishes similar to those achieved in polishing or lapping, are achieved in this high-speed dicing process due to the plastic-flow material removal mechanism. 

## 4. Conclusions

This study presented the benchmarking investigation and analysis of high-speed low-damage micro-cutting of CFRP composites with diamond abrasive blades, particularly in light of obtaining nanoscale smooth surfaces of CFRP composites by introducing nanoscale cutting depth of diamond grits. The paper also presented the dicing mechanism in high-speed micro-cutting of CFRP composites. The surface morphology of the processed specimens with different rotational speeds and fiber orientations were further studied to evaluate and validate the mechanism and analysis. The concluding results are summarized as follows:

(1) Dicing with diamond abrasive blades enables machining CFRP composites with a high cutting speed of more than 76 m/s (25,000 rpm). The high cutting speed not only remarkably increases the rigidity of the ultra-thin dicing blade, but also decreases the cutting depth per diamond grit to below 10 nm. Both result in a fine surface finish of machined CFRP composites. However, the serious vibration of the spindle in the horizontal direction limits the rotational speed to increase further. Best machined surface quality occurs at the rotational speed of 25,000 rpm. The surface roughness of machined CFRP composites have a similar tendency as the cutting current of the machine mainly due to the spindle vibration having a more important influence on the cutting current than the cutting force. Thus, it is necessary to reduce the vibration when the rotational speed is more than 25,000 rpm.

(2) The cutting currents of the spindle motor present an overall V-shape tendency when the fiber orientation angle changes from 0° to 180° due to the mechanical anisotropy of CFRP composites. The maximum value occurs at the fiber orientation of 0°, while the minimum value occurs at the fiber orientation of 90°. With the fiber orientation changing from 0° to 90°, the cutting current presents an overall decrease but a slight increase at about 40°. This tendency is different from most reported research about the milling and drilling of CFRP composites. It may be attributed to the different cutting mechanism of the dicing method with drilling and milling methods. The surface roughness of machined CFRP composites has a similar tendency with the cutting current. When the cutting direction is perpendicular to the fiber orientation, the machined CFRP composite obtains the best surface finish at about Ra 0.025 μm, which is lower than most reported results. Furthermore, the metal-bonded dicing blade achieves smaller surface roughness than the resin-bonded dicing blade when cutting with all fiber orientations due to its good thermal conductivity and low friction coefficient.

(3) In comparison with scratching CFRP composites with the diamond indenter, the mechanism of high machining quality achieved with the dicing blade is mainly attributed to the nanoscale cutting depth of diamond grits. Such a thin cutting layer allows the material removal to only occur at local regions of carbon fiber or resin matrix. The high cutting speed and low cutting force prevent serious deformation or breaking off of bulk fiber. The micro breakage and plastic-flow in local regions are the main material removal mechanisms for dicing CFRP composites with diamond abrasive blades. Thus, the surface roughness of machined CFRP composites mainly depends on the styles of micro breakage. When cutting in the direction perpendicular to the fiber orientation, the surface roughness is lowest due to the well fixation of fibers in resin matrix. However, when cutting in the direction parallel to the fiber orientation, the debonding of fibers causes relative poor machining quality.
